# Cysteine Redox Potential Determines Pro-Inflammatory IL-1β Levels

**DOI:** 10.1371/journal.pone.0005017

**Published:** 2009-03-27

**Authors:** Smita S. Iyer, Carolyn J. Accardi, Thomas R. Ziegler, Roberto A. Blanco, Jeffrey D. Ritzenthaler, Mauricio Rojas, Jesse Roman, Dean P. Jones

**Affiliations:** 1 Nutrition and Health Sciences Program, Emory University, Atlanta, Georgia, United States of America; 2 Division of Endocrinology, Metabolism and Lipids, Emory University, Atlanta, Georgia, United States of America; 3 Division of Pulmonary, Allergy, and Critical Care Medicine, Emory University, Atlanta, Georgia, United States of America; 4 Center for Translational Research in the Lung, Emory University, Atlanta, Georgia, United States of America; 5 McKelvey Center for Lung Transplantation and Pulmonary Vascular Diseases, Emory University, Atlanta, Georgia, United States of America; 6 Clinical Biomarkers Laboratory, Emory University, Atlanta, Georgia, United States of America; 7 Atlanta Veterans Affairs Medical Center, Emory University, Atlanta, Georgia, United States of America; UMR CNRS 5226 - Université Bordeaux 2, France

## Abstract

**Background:**

Cysteine (Cys) and its disulfide, cystine (CySS) represent the major extracellular thiol/disulfide redox control system. The redox potential (E_h_) of Cys/CySS is centered at approximately −80 mV in the plasma of healthy adults, and oxidation of E_h_ Cys/CySS is implicated in inflammation associated with various diseases.

**Methodology/Principal Findings:**

The purpose of the present study was to determine whether oxidized E_h_ Cys/CySS is a determinant of interleukin (IL)-1β levels. Results showed a 1.7-fold increase in secreted pro-IL-1β levels in U937 monocytes exposed to oxidized E_h_ Cys/CySS (−46 mV), compared to controls exposed to a physiological E_h_ of −80 mV (P<0.01). In LPS-challenged mice, preservation of plasma E_h_ Cys/CySS from oxidation by dietary sulfur amino acid (SAA) supplementation, was associated with a 1.6-fold decrease in plasma IL-1β compared to control mice fed an isonitrogenous SAA-adequate diet (P<0.01). Analysis of E_h_ Cys/CySS and IL-1β in human plasma revealed a significant positive association between oxidized E_h_ Cys/CySS and IL-1β after controlling for age, gender, and BMI (P<0.001).

**Conclusions/Significance:**

These data show that oxidized extracellular E_h_ Cys/CySS is a determinant of IL-1β levels, and suggest that strategies to preserve E_h_ Cys/CySS may represent a means to control IL-1β in inflammatory disease states.

## Introduction

Interleukin (IL)-1β is a pro-inflammatory cytokine that functions as a critical regulator of host defense in response to infection and injury. However when present in excess, IL-1β is extremely toxic [1]. Elevated systemic levels of IL-1β cause hypotension during septic shock and induce capillary leak in acute lung injury [2]. IL-1β is also involved in chronic inflammation associated with arthritis, lung fibrosis, and atherosclerosis [3], [4], [5]. Therefore, strategies to modulate IL-1β production in inflammatory diseases are of therapeutic interest.

IL-1β activation and induction are associated with inflammation, a process with enhanced generation of reactive oxygen and nitrogen species [6]. These reactive species serve multiple biological functions, which include removal of cell debris and cell signaling necessary for host defense. Recent advances in redox signaling mechanisms have revealed that functional pathways utilize diffusible oxidants such as peroxide and redox-sensitive thiols in specific proteins as sensors [7]. The redox states of these sensors are controlled by rates of oxidation of specific amino acid residues and their reduction by thiol/disulfide control systems.

The thiol/disulfide control systems are compartmentalized; glutathione/glutathione disulfide (GSH/GSSG) and thioredoxin provide control mechanisms within cells, while cysteine/cystine (Cys/CySS) and GSH/GSSG control the redox state of proteins in the extracellular space and on the cell surface [8]. The Cys/CySS couple predominates in the extracellular fluid and the steady-state redox potential (E_h_) of Cys/CySS is oxidized in acute and chronic inflammatory disease states [9]. *In vitro,* oxidized E_h_ Cys/CySS induces upregulation of nuclear factor-kappa B (NF-κB) [10], [11], increases adhesion of leukocytes to the endothelium [10], and sensitizes epithelial cells to apoptosis [12]. Based on these observations, we hypothesized that extracellular E_h_ Cys/CySS is a determinant of pro-inflammatory cytokine production.

We tested this hypothesis by modifying extracellular E_h_ Cys/CySS and determining IL-1β levels *in vitro* and *in vivo*. *In vitro* results showed that oxidized extracellular E_h_ Cys/CySS is sufficient to increase pro-IL-1β levels in monocytes, and *in vivo* results showed that dietary treatment to protect against plasma E_h_ Cys/CySS oxidation is associated with decreased IL-1β levels in LPS-challenged mice. Analysis of E_h_ Cys/CySS and IL-1β in human plasma revealed a significant positive association between oxidized E_h_ Cys/CySS and IL-1β, independent of age, gender, and BMI. Together, the data show that oxidized extracellular E_h_ Cys/CySS is one of the determinants of IL-1β levels, and suggest that strategies to preserve E_h_ Cys/CySS may represent a means to control IL-1β in inflammatory disease states.

## Materials and Methods

### Ethics Statement

All protocols involving human subjects were reviewed and approved by the Emory Institutional Review Board. All protocols involving mice were reviewed and approved by the Institutional Animal Care and Use Committee at Emory University.

### Materials

Except as indicated, all chemicals were purchased from Sigma Chemical Corporation (Sigma, St. Louis, MO). Distilled, deionized water was used for analytical purposes. HPLC quality solvents were used for HPLC.

### Cell culture

Human monocytic cells (U937, ATCC, Rockville, MD) were maintained in RPMI-1640 supplemented with 10% fetal bovine serum (FBS, Atlanta Biologicals, Norcross, GA) and 10 U/ml penicillin and streptomycin sulfate. Cells were transferred to 0.5% FBS media 8–12 h prior to experimental manipulations.

To generate the desired range of extracellular redox potentials, the extracellular thiol/disulfide pool was altered by varying concentrations of Cys and CySS, added to cyst(e)ine-free RPMI, as previously described [11]. In these experiments, the total extracellular pool size of Cys+CySS was set at 200 µM, while concentrations of Cys and CySS were varied to obtain initial E_h_ values from −80 mV (physiological) to −46 mV (oxidized). The measured Cys/CySS redox range in human plasma is −120 to −20 mV [13]. E_h_ Cys/CySS is centered at −80 mV in healthy individuals and becomes oxidized with age, lifestyle factors such as smoking and alcohol abuse [14], [15], and pathologies such as atrial fibrillation [16], and age-related macular degeneration [9]. Therefore, the experimental range of −80 to −46 mV covers the physiological range and extends to oxidizing values that are reachable under pathological conditions.

Production of reactive oxygen species (ROS) was detected using 6-Carboxy-2′,7′-dichlorofluorescein diacetate (DCFH-DA, Molecular Probes, Eugene, OR, USA) [10]. U937 cells, plated into 96-well plate, were washed with KRH buffer and incubated with 100 µM DCFH-DA for 30 minutes (37°C, 5% CO_2_). Cells were washed and exposed to physiological (−80 mV) and oxidized (−46 mV) Cys/CySS redox media at 37°C. Oxidation of DCFH-DA to fluorescent DCF was measured on a microplate reader (excitation, 485 nm; emission, 530 nm) [17].

### Experimental animals and dietary intervention

Experiments were conducted using 10–14 week old, female C57BL/6J mice (Jackson Laboratories, Bar Harbor, ME). Mice were housed in cages and maintained on a 12-h light-12-h dark cycle at the Division of Animal Resources at Emory University. All experiments were initiated during the light cycle. All animal protocols were reviewed and approved by the Institutional Animal Care and Use Committee.

Prior to the dietary intervention, all animals were fed pelleted rodent food (Test Diet 5015, Lab Diet Inc., Richmond, IN). Semi-purified diets were custom-prepared (Harlan-Teklad, Madison, WI, USA) in order to test the specific effects of sulfur amino acid (SAA) supplementation [18]. The SAA-adequate and SAA-supplemented diets were isocaloric and isonitrogenous and contained adequate and identical quantities of energy, nitrogen, carbohydrate, fat, fiber, micronutrients and essential amino acids. The SAA and nitrogen content was controlled at the desired experimental levels by varying the amount of cystine and methionine and the non-essential amino acids L-alanine, L-aspartic acid, glycine and L-serine. The SAA-supplemented diet contained 3-fold cystine, and 1.8-fold methionine compared to the SAA-adequate diet. Animals had free access to water at all experimental time points.

### LPS Administration

*Escherichia coli* O55:B5 LPS, dissolved in sterile PBS (100 µg/ml) was administered intraperitoneally to unanaesthetized animals at a dose of 1 mg LPS/kg body weight. Animals were sacrificed at 2 h post-LPS. The dose of LPS was used based on previous observations that it substantially increases plasma and lung IL-1β levels [19], and oxidizes plasma E_h_ Cys/CySS at 2 h [20].

### Sample collection and analysis of Cys, CySS, GSH and GSSG

Mice were anesthetized by isofluorane inhalation (Baxter Pharmaceuticals, Deerfield, IL). Opening of the blood brain barrier is reported at high concentrations of isoflurane [21]. The implications of this deleterious effect of isoflurane to measurements made in the current study are presently unknown. Subsequent to anesthesia, blood was collected by submandibular bleeding using a 4 mm mouse bleeding lancet (Medipoint, Inc. Mineola, NY). 0.18 ml of collected blood was immediately transferred to 0.02 ml of preservation solution containing γ-glutamyl-glutamate (γ-Glu-Glu) as an internal standard [22].

Samples were centrifuged at 16000 g for 60 seconds to remove precipitated protein, and 0.1 ml of the supernatant was immediately transferred to an equal volume of ice-cold 10% (w/v) perchloric acid. Samples were immediately stored at −80°C.

For HPLC analysis (Gilson Medical Electronics, Middleton, WI), derivatized samples were centrifuged, and 50 µl of the aqueous layer was applied to the Supercosil LC-NH_2_ column (25 cm×4.6 mm; Supelco, Bellefunk, PA). Derivatives were separated with a sodium acetate gradient in methanol/water and detected by fluorescence [23]. Concentrations of thiols and disulfides were determined by integration relative to the internal standard. Redox potential s (E_h_) of the GSH/GSSG and Cys/CySS pools, given in millivolts (mV), were calculated from concentrations of GSH, GSSG and Cys, CySS in molar units with the following forms of the Nernst equation for pH 7.4: GSH/GSSG, E_h_ = −264+30 log ([GSSG]/[GSH]^2^), Cys/CySS, E_h_ = −250+30 log ([CySS]/[Cys]^2^)[24].

### Quantitative Real-Time PCR Analysis

Lung samples were excised, snap frozen in liquid N_2_ and stored at −80°C. Total RNA was extracted from tissue using an RNeasy Midi Kit (QIAGEN Inc., Valencia, CA) according to manufacturer's instructions. DNase treatment was performed to remove contaminating genomic DNA. RNA concentration was spectrophotometrically determined at 260 nm, and 0.5 µg of total RNA was used to synthesize 20 µl of cDNA (Invitrogen, Carlsbad, CA). Quantitative real-time PCR was performed on cDNA using gene-specific primers on an iCycler IQ Real-Time PCR Detection System (Bio-Rad Laboratories, Hercules, CA). Primers were designed using Beacon Designer Software 4.00 (PREMIER Biosoft International, Palo Alto, CA) (Table 1). Samples containing serial dilutions of known concentrations of cDNA, encoding the gene of interest, were amplified in parallel. Data were analyzed using the iCycler Software, and starting quantities of message levels of each gene were determined from constructed standard curves. Melt curves were examined to ensure amplification of a single PCR product. Expression level of IL-1β was normalized to β-actin in monocytes and 18S ribosomal RNA in mouse lung tissue.

**Table 1:** Details of PCR primer sequences used in the analyses of extracted RNA for quantitative real-time PCR.

Target	Genbank accession number	Forward primer	Reverse primer	Product size, bp
IL-1β human	NM_000576	TGATGGCTTATTACAGTGGCAATG	GTAGTGGTGGTCGGAGATTCG	140
β-actin human	NM_001101	GCGTGACATTAAGGAGAAG	GAAGGAAGGCTGGAAGAG	172
IL-1β mouse	NM_008361	ATCTCGCAGCAGCACATC	CAGCAGGTTATCATCATCATC	192
18S mouse	NR_003278	CGTCTGCCCTATCAACTTTCG	GCCTGCTGCCTTCCTTGG	130

### Cytokine and Western blot analysis

Levels of IL-1β in the cell-supernatant were detected by ELISA (R&D Systems, Minneapolis, MN), and are expressed relative to total protein content in the supernatant. Western Blot analysis of the cell extract from U937 cells was performed to detect the precursor form of IL-1β. After washing with PBS, cells were lysed in homogenization buffer (50 mM NaCl, 50 mM NaF, 50 mM NaP_2_0_7_-10 H_2_0, 5 mM EDTA, 5 mM EGTA, 2 mM Na_3_ V0_4_, 0.5 mM PMSF, 0.01% Triton X-100, 10 ug/ml leupeptin, 10 mM HEPES, pH 7.4) and the concentration of proteins was determined by using a Bradford reagent (Bio-Rad, Hercules, CA). Equal amounts of protein were loaded onto 10% acrylamide SDS/PAGE gradient gels (Bio-Rad).

Proteins were transferred onto nitrocellulose membranes using a semi-dry trans-blot apparatus set at 25 V for 1 h (Bio-Rad). Membranes were subsequently incubated overnight with an anti-human IL-1β polyclonal antibody (Cell Signaling Technology, Danvers MA). Rabbit polyclonal anti-β-actin (Abcam Cambridge, MA) was used as a loading control. After washing with tris buffered saline (TBS) (10 mM Tris-HCL, pH 8.0, 150 mM NaCl, 0.05% Tween-20), membranes were incubated for about 1 h at room temperature with goat anti-rabbit IgG conjugated to horseradish peroxidase-coupled secondary antibody. After further washing, immunoreactive signals were determined by chemiluminescence. Protein bands were quantified by densitometric scanning using a GS-800 Calibrated laser densitometer (Bio-Rad).

To better assess the inflammatory status *in vivo*, levels of IL-1β, and tumor necrosis factor (TNF)-α were measured. IL-1β and TNF-α in human plasma, mouse plasma, and mouse lung homogenates were detected by immunofluorescence using a multiplex panel assay (R&D Systems, Minneapolis, MN). Briefly, the antibody coupled beads were incubated with the sample followed by incubation with a detection antibody. Fluorescence intensity was read on a Bioplex suspension array system (Bio-Rad, Hercules, CA).

### IL-1β luciferase reporter assay

To evaluate IL-1β gene transcription, U937 monocytes were electroporated with a pIL-1 (4.0 kb) luciferase promoter construct, as previously described [25]. Cells were plated in 6-well plate and were incubated for 8 h in specific redox media. Cells were lysed in reporter lysis buffer (Promega, Madison, WI) and assayed for luciferase activity. Measurements were done using a Labsystems Luminoskan Ascent Plate Luminometer. Results were recorded as luciferase units and were adjusted for total protein content.

### Human subjects

This study was reviewed and approved by the Emory Institutional Review Board. A total of 16 healthy volunteers were recruited by posting fliers in public locations in the Atlanta/Emory University community. Following written informed consent, participants were admitted to the outpatient unit of the Emory University Hospital General Clinical Research Center (GCRC), where potential subjects were screened using a medical history, physical examination, urinalysis, standard chemistry profile and a complete blood count. Eligibility was established based on the following criterion: absence of acute or chronic illness (other than a medical history of well-controlled hypertension), BMI<30, non-smokers, and compliance in discontinuing nutritional supplements, if consumed, 2 weeks prior to study entry. Eligible participants were then scheduled for a 24 h inpatient visit within 2 weeks of screening in the GCRC. Characteristics of study subjects are presented in Table 2.

**Table 2:** Characteristics of study subjects.

Demographics	
Age, mean+SD	60.3+17.9
BMI, mean+SD, kg/m^2^	24.2+3.1
Females, %	56

Fasting markers, mean+SD	
Cys, µM	9.2+2.8
CySS, µM	66.6+13.9
E_h_Cys/CySS, mV	(−)71.7+9.2
GSH, µM	1.2+0.4
GSSG, µM	0.07+0.02
E_h_GSH/GSSG, mV	(−)122.8+9
TNF-α, pg/mL	4.5+3.7
IL-1β, pg/mL	3.6+3.6

### Human study protocol

Participants were instructed not to eat after 10 pm the night prior to the inpatient visit in order to standardize baseline levels of metabolites. Participants were admitted to the GCRC at 7.00 on Day 1 and a heparin-lock catheter was placed in a forearm vein for blood sampling at 8.00. After a 30-minute supine resting period, 3 ml blood samples were drawn every hour for 24 consecutive hours. Participants were given breakfast at 9:30, lunch at 13:30, dinner at 17:30, an evening snack was provided at 21:30 immediately following the timed blood draw for that hour. The composition of the meals and snack was standardized for all subjects, as previously reported [26]. Water was provided *ad libitum* throughout the study period.

### Statistical Methods

Data are presented as means+SEM. Statistical analysis was done using SAS v 9.1 (SAS Institute Inc., Cary, NC, US). Data from *in vitro* experiments were analyzed using an unpaired t-test. Data from *in vivo* murine experiments were analyzed using a one-way ANOVA with treatment specified as the main effect. To analyze data from human plasma, a linear mixed model was used. The association of Cys, CySS, and E_h_ Cys/CySS with IL-1β, and TNF-α was determined after controlling for time of day, BMI, age, and gender. Because none of the potential confounders had statistically significant regression coefficients, parameters for BMI, age, and gender were excluded to arrive at the most parsimonious model. The residuals for thiol/disulfide redox were normally distributed; therefore thiol/disulfide redox was specified as the response variable in all constructed models to improve the stability of the computed regression coefficients. The human study by its very design cannot establish a cause-effect relationship; causality is therefore not invoked by specification of thiol/disulfide redox as dependent and cytokine as the independent variable. The best model from all subsets was determined using the Akaike Information Criterion (AIC). Significance was set at a P value <0.05 for all tests.

## Results

### Oxidized extracellular E_h_ Cys/CySS increases pro-IL-1β and IL-1β mRNA levels in human monocytes

Measurements of Cys and CySS in human plasma show that E_h_ Cys/CySS is centered at approximately −80 mV in healthy young individuals [24] and is oxidized in association with inflammatory disease states [9]. *In vitro*, oxidized extracellular E_h_ Cys/CySS activates nuclear factor (NF)-ΚB, a key pro-inflammatory transcription factor [10], [11]. Together, these data indicate that oxidized E_h_ Cys/CySS is likely to modulate pro-inflammatory cytokine production. Because peripheral blood monocytes are constantly exposed to the extracellular redox environment, we determined whether levels of IL-1β, a major monocyte-derived cytokine, are increased by oxidized extracellular E_h_ Cys/CySS.

**Figure 1:**
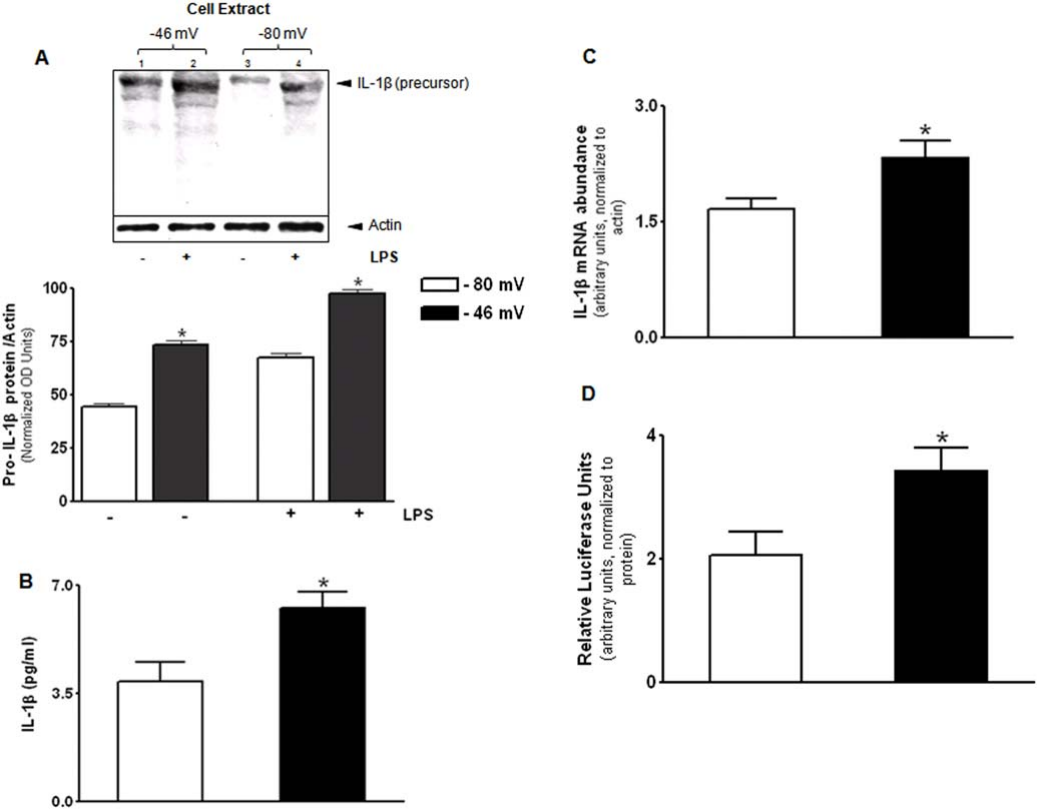
Oxidized extracellular E_h_ Cys/CySS increases pro-IL-1β in monocytes. U937 cells were exposed to physiological (−80 mV) and oxidized (−46 mV) E_h_ Cys/CySS for 8 h and levels of the IL-1β precursor were determined by Western blot (A). Western blot analysis of the cell extract revealed increase in the 31 kDa precursor form of IL-1β at −46 mV compared to –80 mV. Quantitative analysis of the band densities of three separate experiments is shown as a bar graph. In (B) IL-1β levels determined by ELISA are expressed relative to protein concentration in the cell-supernatant. In (C), total RNA was extracted 4 h after treatment with redox media. Abundance of IL-1β mRNA was detected by real-time PCR and is normalized to β actin. In (D), U937 cells expressing the IL-1β-luciferase construct were exposed to given E_h_ for 12 h. Luciferase activity in cell-lysates is shown after normalization for total protein. Data are mean+SE of 3 replicates of a representative experiment repeated 3 times, * P<0.05 between −80 mV and −46 mV treatments.

U937 monocytes were exposed to E_h_ of −80 mV (physiological) or −46 mV (oxidized) for 8 h and levels of IL-1β were determined in the cell extract by western blot using an antibody that detects both the precursor and mature forms of IL-1β. Figure 1A shows an increase in cellular levels of the IL-1β precursor at −46 mV compared to −80 mV (lane 1 versus lane 3; P<0.05). IL-1β levels were also determined in cells treated with LPS (100 µg/ml) under different redox conditions, to assess whether IL-1β is increased in oxidized extracellular E_h_ under inflammatory states. The data show increased cellular levels of the IL-1β precursor at −46 mV compared to −80 mV with LPS treatment (lane 2 versus lane 4; P<0.05).

Because an increase in the precursor form of IL-1β was observed in the cell extract under oxidized conditions, we next attempted to quantify secreted IL-1β levels in the cell-supernatant by ELISA. In monocytes and macrophages, the release of mature IL-1β is dependent on stimulation of caspase-1 by extracellular ATP [27]. Because ATP was not added to the media, the form of IL-1β measured by ELISA is likely the precursor form. Figure 1B shows a 1.7-fold increase in precursor form of IL-1β in cells treated with E_h_ of −46 mV compared to −80 mV (P<0.01).

To determine whether increased pro-IL-1β in response to oxidized E_h_ occurred due to increase in mRNA abundance, IL-1β mRNA was quantified by real-time PCR. As shown in Figure 1C, IL-1β mRNA was more abundant in response to E_h_ of −46 mV, 4 h after treatment (P<0.05). Experiments with U937 cells expressing the human IL-1β promoter fused to a luciferase reporter gene revealed significant increase in luciferase activity at −46 mV, suggesting transcriptional induction of IL-1β by oxidized E_h_ (Figure 1D; P<0.05).

### Oxidized extracellular E_h_ Cys/CySS induces intracellular ROS production in monocytes but has no effect on cellular E_h_ GSH/GSSG

**Figure 2:**
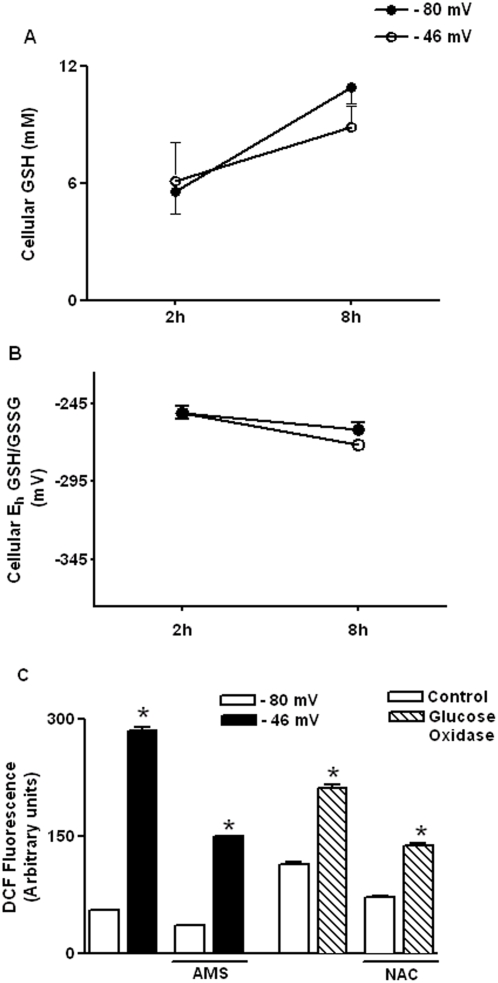
Oxidized extracellular E_h_ Cys/CySS induces ROS production in monocytes but has no effect on cellular E_h_ GSH/GSSG. U937 cells were lysed 2 h and 8 h after exposure to −80 mV and −46 mV Cys redox states. Cellular concentrations of GSH and GSSG were determined by HPLC. GSH levels (A) and E_h_ GSH/GSSG (B) were not significantly different between 80 mV and −46 mV treatments. In (C), U937 cells were pre-incubated with an ROS-sensitive dye, DCFH-DA (100 µM) for 30 min, before treating with −80 mV and −46 mV redox media for 5 min at 37°C. Oxidation of DCFH-DA to fluorescent DCF was measured on a microplate reader. Cells exposed to oxidized Cys/CySS redox (−46 mV) show a 5-fold increase in ROS production compared to cells exposed to a physiological redox potential of −80 mV (*P<0.001). Pre-treating cells with 0.25 mM 4-acetamide-4′-amleimidylstilbene-2,2′-disulfonic acid (AMS), a non-permeant alkylating agent, attenuated the increase in ROS production (P<0.001). As a positive control, ROS production was measured in monocytes treated with glucose oxidase (2 units); an enzyme system that generates H_2_0_2_ (*P<0.001). NAC pre-treatment attenuated glucose oxidase-induced ROS production (*P<0.001). Data are mean+SE of 4 replicates of a representative experiment repeated 3 times.

Previous studies have shown that induction of IL-1β by extracellular stimuli, such as ATP, is associated with generation of ROS and up-regulation of genes involved in GSH synthesis [27]. Therefore, we determined whether increase in IL-1β in response to oxidized extracellular E_h_ Cys/CySS is associated with intracellular redox changes. Measurement of cellular GSH (Figure 2A) and E_h_ GSH/GSSG (Figure 2B) revealed no significant differences between the −80 and −46 mV treatment groups at 2 h and 8 h.

Next, we examined whether oxidized extracellular E_h_ Cys/CySS stimulated cellular ROS production. Cells were pre-incubated with a ROS-sensitive dye, DCFH-DA, prior to treatment with −80 mV and −46 mV Cys/CySS redox media. Oxidation of DCFH-DA to fluorescent DCF was measured on a microplate reader. Cells treated with glucose oxidase served as positive controls. Within 5 minutes, cells exposed to oxidized E_h_ of −46 mV showed a 5-fold increase in DCF fluorescence compared to cells exposed to a physiological redox potential of −80 mV (P<0.001) (Figure 2C). When measured 2–6 h after treatment, the magnitude of DCF fluorescence increased in both −80 and −46 mV conditions, with at least a 2-fold higher fluorescence at −46 mV (P<0.001, data not shown). This indicates that oxidized E_h_-induced ROS production was sustained over-time, and occurred in the absence of oxidation to cellular E_h_ GSH/GSSG.

We subsequently determined whether increase in ROS, in response to oxidized extracellular E_h_ Cys/CySS, was sensitive to the oxidation of redox-sensitive membrane-bound thiols. To this end, monocytes were pre-treated with 0.25 mM 4-acetamide-4′-amleimidylstilbene-2,2′-disulfonic acid (AMS), a non-permeant alkylating agent, prior to exposing the cells to given E_h_ Cys/CySS. Pre-treatment with AMS decreased (P<0.001), but did not completely inhibit, cellular ROS levels in response to oxidized E_h_ indicating that increase in cellular ROS occurs in part due to oxidation of membrane-bound thiols [10].

Taken together the *in vitro* data show that oxidized extracellular E_h_ Cys/CySS induces a rapid and sustained increase in ROS in monocytes compared to physiological E_h_, which could be involved in oxidized E_h_ mediated increase in IL-1β. Based on these *in vitro* findings, we wanted to determine whether increase in IL-1β during inflammation *in vivo* can be attenuated by preservation of E_h_ Cys/CySS from oxidation.

### Dietary SAA supplementation protects against endotoxin-induced perturbations in plasma Cys, CySS and E_h_ Cys/CySS

Based on previous research showing that supplementation with sulfur amino acids (SAA) increases plasma Cys and shifts the E_h_ to a more reduced potential [18], we applied this approach to a mouse model of inflammation in which LPS/endotoxin is administered to increase IL-1β [19]. In this design, mice were fed either a SAA-adequate diet or an isonitrogenous SAA-supplemented diet for 7 days prior to endotoxin challenge. Mice were sacrificed 2 h post-endotoxin, a time point that coincides with maximal IL-1β production [19]. Plasma was collected prior to sacrifice for analysis of Cys, CySS, GSH and GSSG by HPLC. Thiol/disulfide redox parameters in untreated controls, fed pelleted rodent chow, are shown for comparison. Redox potential values for GSH and Cys are approximately 10–15 mV more reduced in mice plasma compared to human plasma.

**Figure 3:**
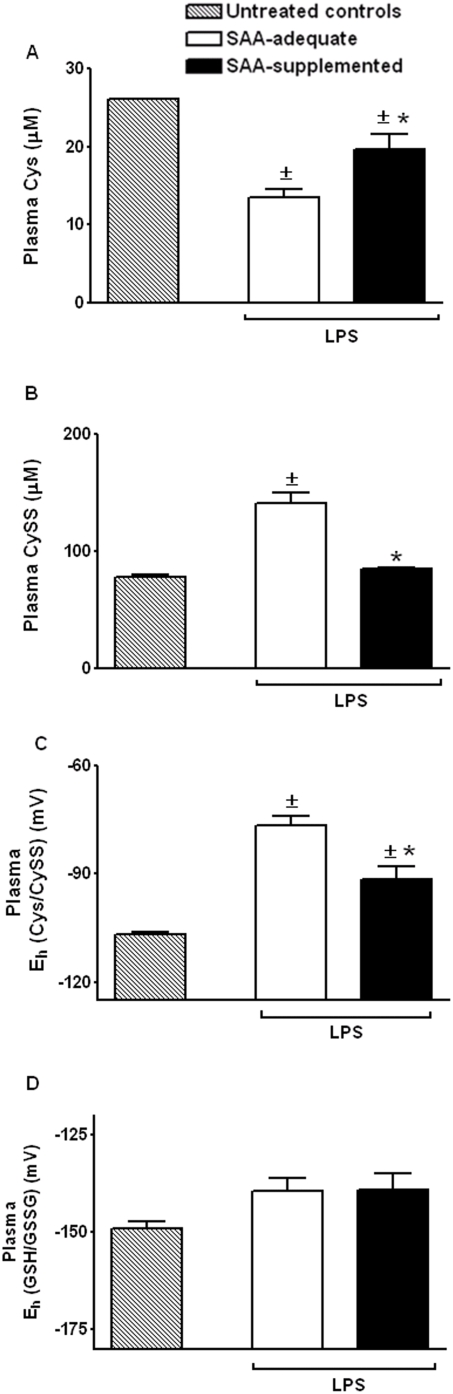
Effect of dietary SAA-supplementation on endotoxin-induced oxidation of plasma E_h_ Cys/CySS. C57BL/6J mice receiving either SAA-adequate diet or SAA-supplemented diet were treated with 1 mg/kg i.p endotoxin/LPS. At 2 h, mice were sacrificed and plasma was collected for HPLC analysis of Cys (A), CySS (B). In (C), E_h_ Cys/CySS was calculated from Cys and CySS concentrations using the Nernst equation. Plasma E_h_ GSH/GSSG is shown in (D). Data are mean+SE of 4 replicates of a representative experiment repeated 2 times. ±Values significantly different from untreated controls, *Values significantly different from SAA-adequate group.

SAA-supplementation protected against endotoxin-induced decrease in plasma Cys (Figure 3A; Cys (µM) - SAA supplemented, 19.6±1.9; SAA-adequate, 13.5±1.2; P<0.05). The decrease in plasma Cys in response to endotoxin is likely due to clearance of Cys, and oxidation of Cys to CySS. *Ex vivo* studies in hepatic membrane vesicles show that LPS stimulates hepatic influx of Cys to support gluconeogenesis and synthesis of acute phase proteins [28]. LPS also increases peroxide levels [29], and this could decrease plasma Cys via oxidation to CySS, an interpretation supported by the increase in plasma CySS levels in mice fed SAA-adequate diet (Figure 3B). However, because the magnitude of increase in CySS exceeds the decrease in Cys, mechanisms related to altered transport or recycling of Cys and/or CySS may also contribute to alterations in Cys/CySS homeostasis during endotoxemia.

The difference in plasma Cys between SAA-supplemented and SAA-adequate group is consistent with the predicted increase in Cys due to excess SAA intake, based on previous work done in our laboratory [18]. However, plasma CySS levels were not affected by excess SAA intake [18]. Thus, the lack of increase in plasma CySS in the SAA-supplemented group, 2 h post endotoxin, suggests additional effects on ROS homeostasis due to SAA supplementation (CySS (µM) - SAA-adequate, 141±9.7; SAA supplemented, 85.2±1.2; P<0.01). As a consequence of the higher Cys and decreased CySS, E_h_ Cys/CySS was on average 15 mV more reduced in response to endotoxin in the SAA-supplemented group compared to SAA-adequate group (Figure 3C; SAA-adequate, −76.4±2.6; SAA supplemented, −91.5±3.7; P<0.01).

Measurements of plasma GSH, GSSG (data not shown) revealed that plasma E_h_ GSH/GSSG (Figure 3D) was not oxidized after endotoxin treatment, and was comparable across treatment groups. These results confirm that the *in vivo* model is adequate to test whether attenuating oxidation of plasma E_h_ Cys/CySS decreases plasma IL-1β levels in inflammation.

### Dietary SAA supplementation protects against endotoxin-induced IL-1β and TNF-α

**Figure 4:**
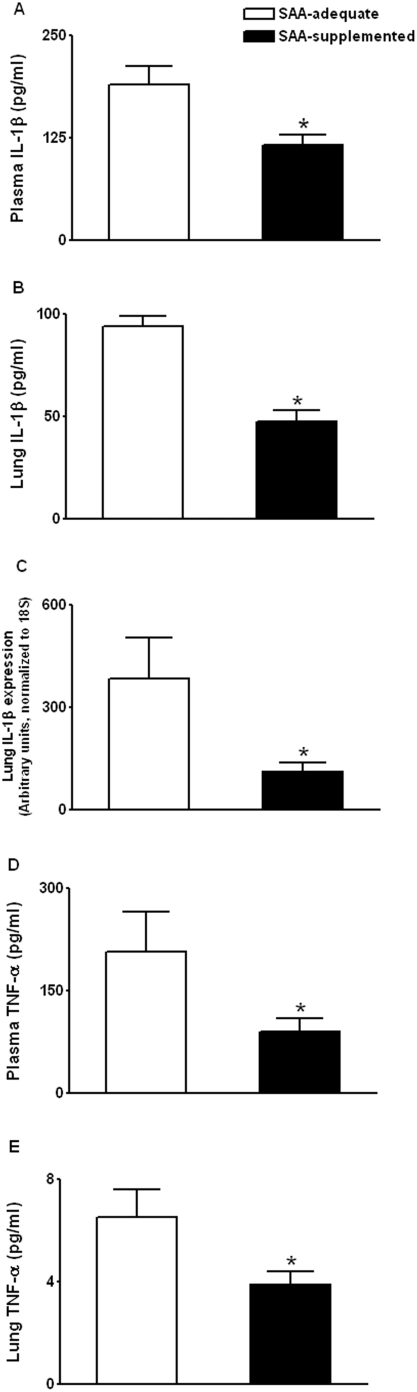
Effect of dietary SAA-supplementation on endotoxin-induced IL-1β and TNF-α. C57BL/6J mice receiving either SAA-adequate diet or SAA-supplemented diet were treated with 1 mg/kg i.p endotoxin/LPS. At 2 h, mice were sacrificed and plasma and lung samples were collected for analysis of IL-1β and TNF-α. Plasma IL-1β levels are shown in (A). IL-1β levels in lung homogenate are presented after normalization for total protein (B). In (C) RNA was extracted from whole lung and transcript levels of IL-1β were quantified by quantitative real-time PCR. Plasma TNF-α (D) and lung TNF-α (E) were also determined. Data are mean+SE of 4 replicates of a representative experiment repeated 2 times, * P<0.05.

Next, we determined whether the more reducing plasma E_h_ Cys/CySS, in SAA-supplemented animals is associated with a decrease in plasma IL-1β, in response to endotoxin. Plasma IL-1β levels in untreated controls were un-detectable (not shown). As shown in Figure 4A, plasma IL-1β decreased by 1.6-fold in SAA-supplemented animals (IL-1β (pg/ml) - SAA adequate, 190±22; SAA supplemented, 116±12; P<0.01). Induction of IL-1β in the lung is an early response to endotoxin [20], so we determined whether a protective effect on IL-1β was also observed in the lung, in response to a reduced extracellular E_h_ Cys/CySS. Lung IL-1β levels were decreased by 2-fold in the lung homogenate from the SAA-supplemented group (Figure 4B; P<0.001). Measurements of IL-1β message levels by quantitative real-time PCR revealed a 3-fold decrease in lung IL-1β mRNA levels (Figure 4C; P<0.05). Thus, preserving extracellular E_h_ Cys/CySS during endotoxemia decreased tissue and circulating levels of IL-1β.

Tumor necrosis factor (TNF)-α is another prototypical pro-inflammatory cytokine that is induced in concert with IL-1β in response to infection, injury, and immunological challenge [30]. To better assess the inflammatory status *in vivo*, we determined whether attenuation of TNF-α also occurred in SAA-supplemented mice. Results showed a greater than 2-fold decrease in plasma TNF-α levels with SAA-supplementation (Figure 4D; P<0.05). TNF-α in the lung was also significantly decreased (Figure 4E; P<0.05). Thus, the *in vivo* observations show that preservation of plasma E_h_ Cys/CySS during endotoxemia is associated with a decrease in IL-1β levels. These data extend the *in vitro* observations, and the combined findings support a mechanistic role for Cys redox potential in determining IL-1β levels.

### IL-1β and TNF-α in plasma of healthy adults are increased in association with oxidized E_h_ Cys/CySS

To investigate whether Cys redox potential could represent a determinant of pro-inflammatory cytokine levels in humans, we examined IL-1β, TNF-α, Cys, and CySS in plasma samples from 16 healthy adults. The characteristics of the study participants are shown in Table 2.

Because plasma Cys and E_h_ Cys/CySS, and cytokines exhibit well documented circadian rhythms [26], [31], the study was designed to examine whether diurnal variation in E_h_ Cys/CySS was associated with variation in IL-1β and TNF-α. For this purpose, hourly samples were collected for an entire 24 h period from 16 individuals. Diurnal variation in plasma Cys and E_h_ Cys/CySS was closely related to meal intake, as reported previously (data not shown) [26]. However, due to considerable inter-individual variation in plasma IL-1β and TNF-α, we did not detect significant time-dependent variations in cytokine levels. Therefore, we used a secondary analysis to determine whether variation in E_h_ Cys/CySS correlated with variation in IL-1β, independent of time of day.

Because repeated measures were obtained from the same individual, we used a linear mixed procedure to model variation in redox parameters with cytokine levels controlling for time of day, BMI, age, and gender. As none of the potential confounders had statistically significant regression coefficients, parameters for BMI, age, and gender were excluded to arrive at the most parsimonious model. Cys, CySS and E_h_ Cys/CySS were specified as response variables in the analyses because the residuals for these biomarkers were normally distributed. Regression coefficients for the mixed model are presented in Table 3.

**Table 3:** Mixed model of redox parameters and IL-1β and TNF-α controlling for time of day.

Biomarker	Regression coefficients		P
	**IL-1β**	**SE**	
Cys, µM	−0.19	0.09	<0.05
CySS, µM	0.28	0.44	ns
E_h_Cys/CySS, mV	1.08	0.28	<0.001
	**TNF-α**	**SE**	
Cys, µM	−0.06	0.04	ns
CySS, µM	0.41	0.17	<0.05
E_h_Cys/CySS, mV	0.25	0.11	<0.05

**Figure 5:**
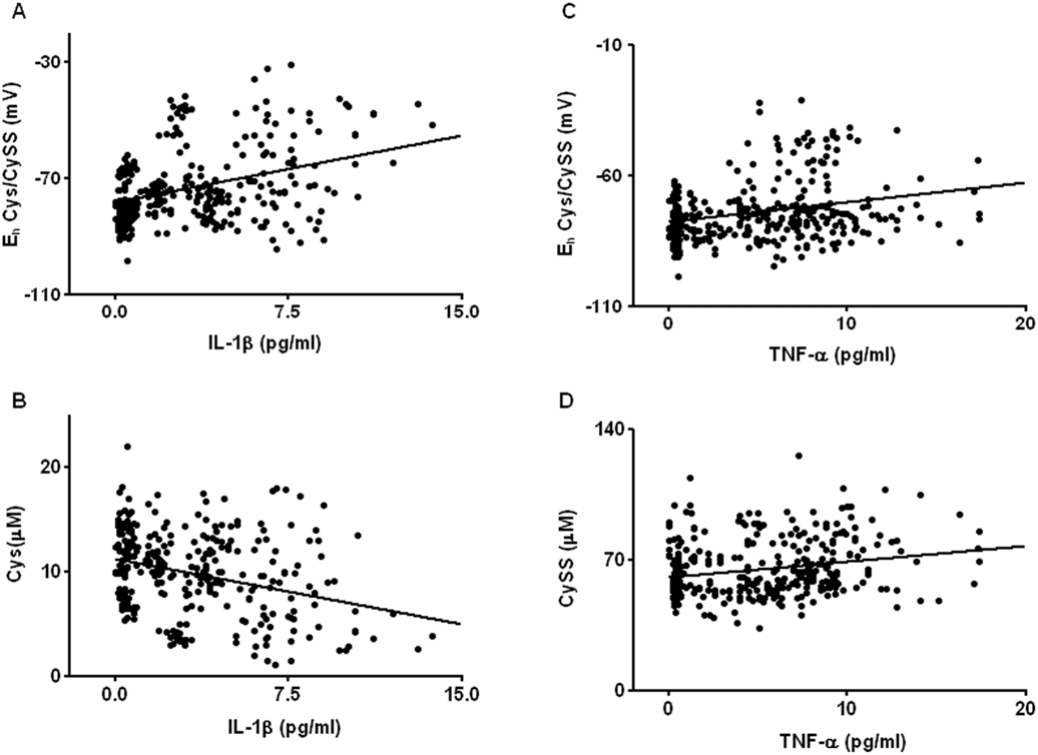
IL-1β and TNF-α in plasma of healthy adults is increased in association with oxidized E_h_ Cys/CySS. A linear mixed model was used to model variation in plasma E_h_ Cys/CySS and IL-1β and TNF-α. A strong positive association was observed between E_h_ Cys/CySS and IL-1β (A; P<0.001). Plasma Cys was negatively associated with IL-1β (B; P<0.05). TNF-α was positively correlated with E_h_ Cys/CySS (C), and CySS (D) (P<0.05).

Examination of redox parameters for Cys redox potential revealed a strong positive association between E_h_ Cys/CySS and IL-1β (P<0.001). As seen in Figure 5A, oxidized values of E_h_ Cys/CySS correlate with higher levels of plasma IL-1β. The regression parameter for IL-1β indicates that a 1 unit increase in plasma IL-1β is associated with a 1.1 mV oxidation of plasma E_h_ Cys/CySS. Analysis of the association between plasma Cys and IL-1β revealed a significant negative association indicating that low levels of plasma Cys correlate with high IL-1β levels (Figure 5B; P<0.05). No significant correlation was observed between plasma CySS and IL-1β. While an association does not establish causality, together with our *in vitro* and *in vivo* data the present findings strongly suggest that Cys redox potential is an important determinant of IL-1β. Plasma TNF-α was also significantly associated with E_h_ Cys/CySS (Figure 5C) and CySS levels (Figure 5D) (P<0.05) suggesting that oxidized Cys redox potential is also a determinant of TNF-α. It must be noted that a caveat that may limit extrapolation of the results to the general population is the age (62 years) and the BMI (24 kg/m^2^) of the population studied. Because old age and overweight are risk factors for inflammation [32], [33], the association between E_h_ Cys/CySS and IL-1β and TNF-α may be less-strong in a younger population with an optimum BMI range.

## Discussion

The current study has three main findings: that oxidized extracellular E_h_ Cys/CySS is sufficient to induce pro-IL-1β in a monocyte cell line; that preservation of plasma E_h_ Cys/CySS from oxidation during endotoxin-induced inflammation is associated with a decrease in circulating IL-1β levels in mice; and that oxidized E_h_ Cys/CySS is positively associated with circulating IL-1β levels in healthy humans. Together, these data suggest that plasma Cys redox potential is not only a biomarker of oxidative stress, but may also be a determinant of immune cell function. Because a number of dietary and behavioral risk factors for disease are known to oxidize Cys redox potential e.g., sulfur amino acid deficiency [18], alcohol [34], and smoking [14]; this provides a mechanistic basis to consider monitoring Cys redox potential as a risk factor for pro-inflammatory states, and consider interventional strategies to control oxidation of Cys redox potential in pro-inflammatory diseases.

Previous studies have shown that induction of IL-1β by non-infectious extracellular stimuli, such as ATP and cigarette smoke condensate, occurs by activation of membrane-bound receptors [27], [35]. The mechanism by which oxidized extracellular E_h_ Cys/CySS induces pro-IL-1β is unknown, but likely involves the generation of intracellular ROS signaled, in part, via oxidation of membrane-bound thiols. Studies in endothelial cells have revealed increased oxidation of cell-surface protein thiols, induction of intracellular ROS, and activation of NF-ΚB in response to an oxidizing extracellular E_h_ Cys/CySS [10]. The rapid and sustained induction of cellular ROS by oxidized E_h_ in the present study is consistent with the same type of mechanism in U937 cells, i.e., the increase in IL-1β by oxidized E_h_ could be signaled via the membrane and involve ROS-dependent activation of pro-inflammatory transcription factors such as NF-ΚB. Interestingly, the increase in ROS by oxidized E_h_ occurred in the absence of changes to intracellular E_h_ GSH/GSSG. This observation is, however, not unexpected. The major thiol/disulfide control systems GSH, thioredoxin, and Cys exist under non-equilibrium conditions and their redox states are distinctly modified by oxidative stress [14], substrate availability [18], [36], [37], physiological [26] and pathological stimuli [8], [36]. Indeed, the present research shows that the oxidation of plasma E_h_ Cys/CySS during acute endotoxemia *in vivo* occurs without the oxidation of plasma E_h_ GSH/GSSG.

The acute oxidation of E_h_ Cys/CySS by LPS is attenuated in mice supplemented with sulfur amino acids (SAA). This effect could be solely due to dietary augmentation of Cys reserves, an interpretation supported by the 1.4-fold higher plasma Cys concentrations in SAA-supplemented mice. However, plasma CySS does not increase in response to LPS suggesting the additional effects on ROS homeostasis due to SAA supplementation. The more reduced redox potential of the Cys/CySS couple in SAA-supplemented mice is associated with a significant decrease in circulating and tissue levels of IL-1β and TNF-α. These data suggest that Cys and associated Cys redox potential are critical determinants of cytokine production during activation of the immune system by LPS in mice. Thus, preservation of E_h_ Cys/CySS by SAA supplementation may be involved in decreased IL-1β and TNF-α levels during endotoxemia. It must be noted, however, that the effects of SAA supplementation may also include preservation of intracellular thiol/disulfide redox status.

In human nutrition, Cys is a conditionally essential amino acid because Cys requirements are normally met by the transulfuration of dietary methionine [38]. However, Cys requirements increase during infection, injury, and in conditions associated with limited hepatic transulfuration [39], [40]. Because Cys is not routinely added to solutions used in parenteral therapy, patients with sepsis could be particularly susceptible to a deficiency of Cys. Studies in humans have shown that plasma Cys redox potential is modulated fairly rapidly by precursor availability [26]. Furthermore, in the present study we find that plasma Cys in humans in negatively associated with plasma IL-1β levels. Thus, nutritional supplementation with Cys or Cys precursors during early sepsis may be a strategy to alleviate acute inflammation and associated tissue injury.

In addition to the pathology associated with disregulated cytokine production in conditions such as sepsis; elevated cytokine levels in healthy individuals independently predict risk of chronic diseases such as type II diabetes and atherosclerosis [41], [42]. Therefore, the association between oxidized E_h_ Cys/CySS and IL-1β and TNF-α in otherwise healthy individuals suggests that oxidized E_h_ Cys/CySS may represent a risk factor for chronic inflammatory diseases. Accordingly, maintenance of Cys redox potential may be critical in protecting against subclinical inflammation in healthy individuals, and in ameliorating pathological processes associated with chronic inflammation. Large population studies with detailed measurements of known factors affecting redox potential along with pro-inflammatory cytokine markers and disease risk factors are needed to test this concept. Such data are critical because assays are available to assess plasma Cys redox potential in humans [22], [43], and simple and inexpensive interventional strategies are available which could improve Cys redox potential [9], [44].

The association between oxidative stress and inflammation is well-recognized and multiple studies have shown that antioxidants such N-acetyl cysteine, have anti-inflammatory effects. The present observations identify a mechanistic link between oxidative stress and inflammation. The combined *in vitro* and *in vivo* observations show that oxidized extracellular E_h_ Cys/CySS is a previously unrecognized modulator of IL-1β. The findings suggest that strategies to preserve E_h_ Cys/CySS may represent a means to control IL-1β in inflammatory disease states.
